# Nitrogen content estimation of apple trees based on simulated satellite remote sensing data

**DOI:** 10.3389/fpls.2025.1613487

**Published:** 2025-07-17

**Authors:** Meixuan Li, Xicun Zhu, Xinyang Yu, Cheng Li, Dongyun Xu, Ling Wang, Dong Lv, Yuyang Ma

**Affiliations:** ^1^ College of Resources and Environment, Shandong Agricultural University, Tai’an, China; ^2^ National Engineering Research Center for Efficient Utilization of Soil and Fertilizer Resources, Shandong Agricultural University, Tai’an, China

**Keywords:** Landsat-8, Sentinel-2, GF-6, nitrogen estimation, phenological period, apple tree

## Abstract

**Introduction:**

Using satellite remote sensing technology to diagnose apple tree nitrogen content is critical for guiding regional precision fertilization of apple trees. However, due to differences in spatial resolution and spectral response, there is a lack of systematic evaluation of satellite data's applicability and accuracy in apple tree nitrogen inversion.

**Methods:**

This study used apple orchards in Qixia City, Shandong Province as the research area, collecting canopy hyperspectral data through an ASD spectrometer during three key phenological periods: the new-shoot-growing stage (NGS), the new-shoot-stop-growing stage (NSS), and the autumn shoot-growing stage (ASS). The data was resampled based on satellite sensor spectral response functions to match the band resolutions of multiple satellite sources. Correlation coefficient method and partial least squares regression were used to screen sensitive bands for apple tree nitrogen content. Support Vector Machine (SVM) and Backpropagation Neural Network (BPNN) algorithms were used to construct and screen the optimal models for apple tree nitrogen content estimation.

**Results:**

Results showed that visible light, red edge, near-infrared, and yellow edge bands were sensitive bands for estimating apple tree nitrogen content. The support vector machine model constructed based on Sentinel-2 satellite simulated data was the optimal nitrogen content inversion model, with an average R^²^ value of 0.81 and an average RMSE value of 0.15 for training sets across different phenological periods, and an average R² value of 0.61 and an average RMSE value of 0.23 for validation sets.

**Discussion:**

This study systematically evaluated the applicability and accuracy differences of multi-source satellite data for estimating nitrogen content in apple trees, and clarified the variation patterns of nitrogen-sensitive spectral bands and optimal modeling strategies across key phenological stages. This research provides a scientific basis for data selection and a technical paradigm for remote sensing-based nutrient diagnosis of apple trees at the regional scale, and holds significant theoretical and practical value for developing region-wide precision fertilization systems based on remote sensing.

## 1 Introduction

Nitrogen is a key nutritional element for apple tree growth, development, and apple quality, with its content changes directly affecting photosynthetic efficiency, leaf area index, and final yield (Li W, et al., 2022; Zhang et al., 2023). Traditional nitrogen diagnostic methods mainly rely on laboratory chemical analysis, such as the Kjeldahl method and spectrophotometric method. Although these methods have high measurement accuracy, they have disadvantages such as strong sample destruction, time-consuming processes, and high costs, making it difficult to meet the needs for real-time and efficient monitoring.

With the rapid development of remote sensing technology, its application in crop growth monitoring, nutrient diagnosis, and yield prediction has demonstrated significant advantages (Goffart et al., 2022; Wen et al., 2024; Zydlik et al., 2024). Depending on the sensor platform, remote sensing can be categorized into proximal sensing, unmanned aerial vehicle (UAV) remote sensing, and satellite remote sensing. Proximal sensors, such as the ASD FieldSpec series, can acquire hyperspectral data with a spectral resolution of 1–3 nm, enabling precise estimation of nitrogen content at the individual plant scale due to their rich spectral information and high estimation accuracy. However, they are limited by their point-based sampling nature and are thus only suitable for small-scale studies (Wang et al., 2019; Li C, et al., 2023). In contrast, UAV-based remote sensing, equipped with multispectral or hyperspectral cameras, offers high spatial resolution (at the centimeter level) and flexible deployment, allowing full orchard coverage and enabling effective nitrogen monitoring in small to medium-sized areas (Wang et al., 2023; Jiang et al., 2025). Nevertheless, its applicability is still constrained by flight altitude and battery life, typically limiting its use to experimental plots of only a few hectares (Li Y, et al., 2023).

At the regional scale, satellite remote sensing has shown great potential in monitoring crop growth and nutrient dynamics due to its wide spatial coverage, short revisit periods, and convenient data acquisition (Jiang et al., 2023; Fu et al., 2024). High-resolution commercial satellites such as RapidEye and WorldView have achieved favorable results in vegetation nutrient monitoring (Magney et al., 2017; Brinkhoff et al., 2019; Wu et al., 2019). However, their high data acquisition costs limit widespread use in routine agricultural management (Huang et al., 2017; Solano et al., 2019; Sozzi et al., 2021). Consequently, the exploration of open-access, cost-effective, and efficient satellite data for monitoring nitrogen content in fruit trees has become a research hotspot.

Currently, widely used open-access satellite data include Landsat-8, Sentinel-2, and GF-6, which have shown great promise in agricultural monitoring (Wolters et al., 2021; Dehghan-Shoar et al., 2023). Landsat-8 is equipped with the Operational Land Imager (OLI), which contains nine multispectral bands, including a near-infrared band (Band 5: 845–885 nm) commonly used for retrieving vegetation chlorophyll and nitrogen content. Croft et al. (2020) found that the normalized difference vegetation index (NDVI) derived from Landsat-8 imagery could effectively estimate chlorophyll content in cropland. Sentinel-2, with its Multispectral Instrument (MSI), includes three red-edge bands (B5: 705 nm, B6: 740 nm, B7: 783 nm), which have proven useful for monitoring vegetation growth and nutrient status (Bossung et al., 2022; Li et al., 2022; Lapaz Olveira et al., 2023). Wang et al. (2025) used Sentinel-2 data in combination with biochemical trait models to achieve high-precision estimation of leaf nitrogen content in almond orchards, thereby providing valuable support for precision agriculture. The GF-6 satellite introduced additional spectral bands such as a yellow-edge band (Band 5: 520–600 nm) and two red-edge bands, further enhancing the capability for early stress detection and nutrient diagnosis. Chen and Xu (2024) demonstrated the potential of GF-6 red-edge bands by constructing NDRE1 and CIred-edge indices, which successfully monitored forest health conditions.

Although these open-access satellite data have achieved promising results in crop nutrient monitoring, their application in fruit trees-particularly in economically significant crops such as apples—remains limited. Direct comparative studies using different satellite sensors to monitor nitrogen content in apple orchards face several challenges, including difficulties in acquiring temporally synchronized imagery over the same region, disparities in spatial resolution (30 m for Landsat-8, 10–20 m for Sentinel-2, and 16 m for GF-6), and differences in spectral band configurations. These factors can introduce systematic errors and complicate the integration and comparison of multi-source satellite data.

To address these challenges, this study simulates satellite reflectance using ground-based canopy hyperspectral measurements and the spectral response functions of three satellite sensors to systematically evaluate the performance of Landsat-8, Sentinel-2, and GF-6 in estimating nitrogen content of apple trees at different phenological stages. Sensitive spectral bands were identified through correlation analysis and partial least squares regression. Subsequently, support vector machine (SVM) and back-propagation neural network (BPNN) models were constructed to determine the optimal nitrogen estimation models and the most suitable monitoring satellite. The objective of this study is to provide a theoretical basis and practical reference for the efficient application of open-access satellite data in fruit tree nutrient monitoring and precision orchard management.

## 2 Materials and methods

### 2.1 Study area

**Figure 1:**
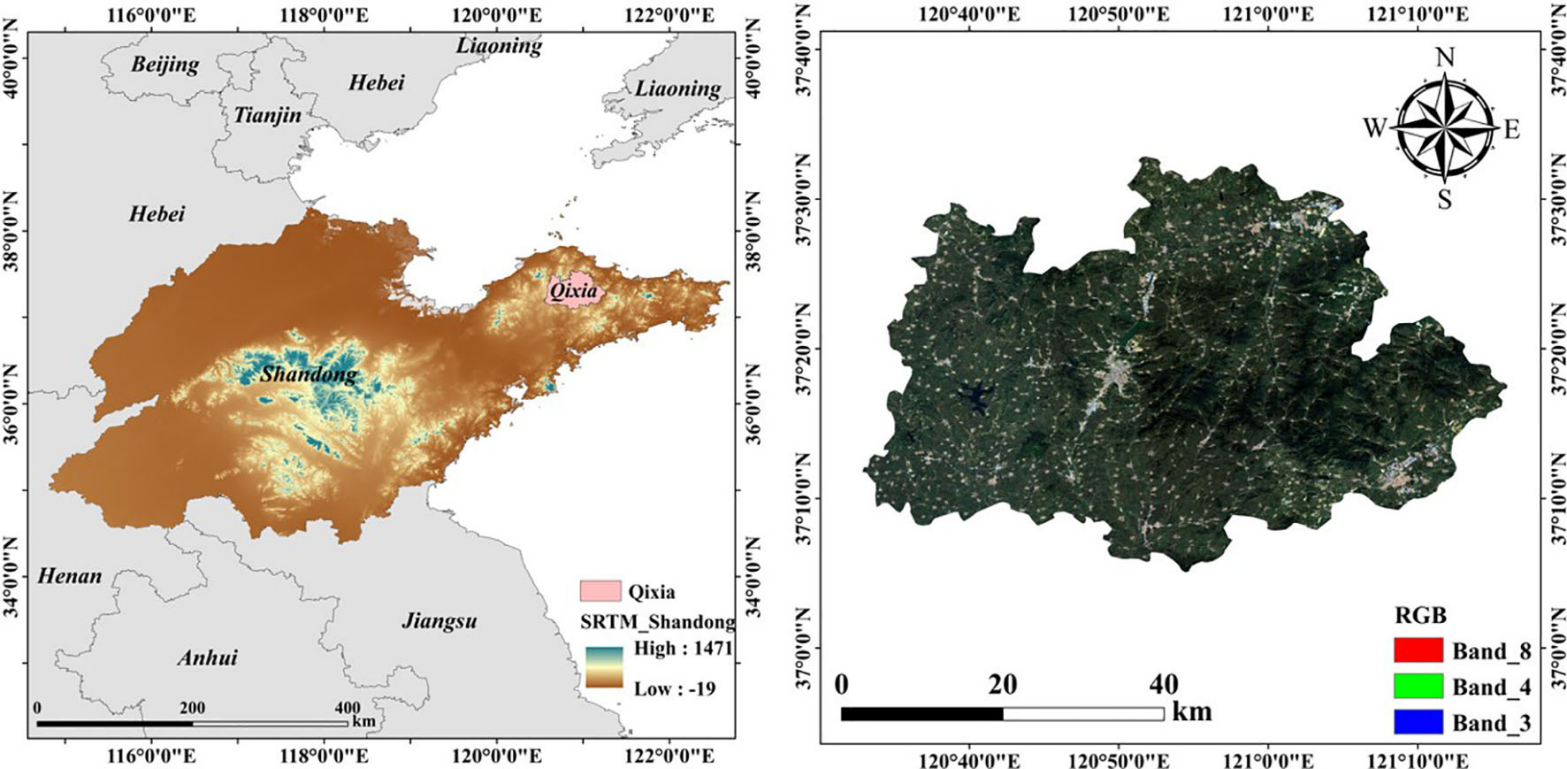
Location map of the study area.

The research area is located in Qixia City, Yantai, Shandong Province, China (37°18’-37°32’N, 121°20’-121°34’E), as shown in **Figure 1**. This region has a warm temperate monsoon humid climate with distinct seasons and large day-night temperature differences, which is beneficial for apple sugar accumulation. The soil type is cambisol, which has good water retention capacity and is very suitable for apple tree growth. Qixia City is known as the “Apple Capital of China” and is one of China’s main apple production areas. The apple orchards in this region are concentrated in distribution, with a wide total area and relatively uniform varieties, providing favorable conditions for large-scale estimation research using remote sensing technology.

### 2.2 Data acquisition and preprocessing

#### 2.2.1 Apple tree leaf sample collection and nitrogen content determination

Using ‘Red Fuji’ apple trees in their apple-bearing period as the research subjects, leaf samples were collected during three key phenological stages in 2023: the new-shoot-growing stage (NGS) on May 19, the new-shoot-stop-growing stage (NSS) on June 20, and the autumn shoot-growing stage (ASS) on September 22. Twenty orchards were randomly selected in the research area, with 5 apple trees randomly chosen from each orchard. From each apple tree canopy, 3 healthy leaves were randomly collected from the east, west, south, and north directions, totaling 12 leaves as one sample. A total of 100 samples were collected for each phenological period. The collected samples were placed in a thermos box and taken back to the laboratory, and the Kjeldahl method was used to determine the nitrogen content of the canopy leaves.

#### 2.2.2 Determination and preprocessing of apple tree canopy hyperspectral data

Canopy reflectance spectra of apple trees were collected using an ASD FieldSpec 4 spectroradiometer (Analytical Spectral Devices Inc., Boulder, CO, USA), which covers a spectral range of 350–2500 nm. The acquisition time is consistent with the collection time of apple leaves. The spectrometer offers a spectral resolution of 3 nm in the visible and near-infrared (VNIR) region around 700 nm and 10 nm in the shortwave infrared (SWIR) regions around 1400 nm and 2100 nm. Reflectance data were recorded at 1 nm intervals, resulting in a total of 2151 continuous spectral bands.

Spectral measurements were conducted under clear, cloud-free conditions between 10:00 and 14:00 local time, when the solar elevation angle exceeded 45°, to minimize the influence of changing illumination. The instrument was preheated for 15 minutes prior to data acquisition. A calibrated white reference panel was used for spectral calibration before each measurement, and optimized every 15 minutes. During optimization, the panel was placed horizontally and kept free of direct shadows to ensure an ideal reflectance value of 1. During measurements, the operator faced the sun to avoid casting shadows on the target. The spectrometer probe was positioned vertically downward above the center of the tree canopy at a height of 1.5 to 3 meters, adjusted according to the crown size to ensure the entire canopy was within the field of view and to reduce interference from canopy structure and shadows. Ten measurements were taken for each observation plot, and the average reflectance value was used to represent the sample. The acquired canopy hyperspectral data were smoothed using preprocessing techniques to reduce noise and improve signal quality. Finally, 100 apple canopy spectral data were obtained for each phenological period.

### 2.3 Research methods

#### 2.3.1 Extraction of different satellite apple tree canopy spectral simulation data

To evaluate the potential of Landsat-8, Sentinel-2, and GF-6 sensors for estimating nitrogen content in apple tree canopies, this study generated simulated satellite data based on ground-measured hyperspectral data. Specifically, the spectral reflectance data collected by the ASD FieldSpec spectrometer were resampled using a convolution operation according to the spectral response functions (SRFs) of the three satellite sensors, producing equivalent satellite band reflectance data under ideal conditions without atmospheric interference (Prey and Schmidhalter, 2019). This approach allows for precise assessment of the sensitivity of different sensor bands to apple tree nitrogen content in an ideal environment, thereby providing theoretical support for the selection of sensitive bands and optimization of modeling procedures prior to the application of actual satellite imagery.

**Figure 2:**
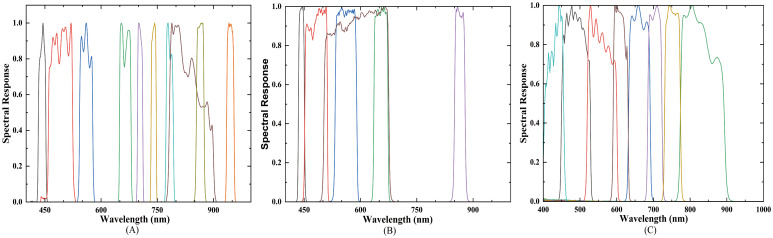
Simulated spectral response functions of satellite sensors. **(A)** Landsat-8; **(B)** Sentinel-2; **(C)** GF-6.

Considering that plant spectral characteristics in the visible region are mainly influenced by chlorophyll content, the near-infrared region is closely associated with leaf internal structure, and the shortwave infrared region predominantly reflects leaf water content variations. Given that nitrogen is a fundamental component of chlorophyll present in both chlorophyll a and b, this study concentrated the resampling on bands within the visible and near-infrared regions to more effectively capture the spectral responses related to nitrogen content. The spectral response functions (SRFs) employed for resampling are presented in **Figure 2**, while the key parameters of the three sensors are summarized in **Tables 1**–**3**. The spectral resampling procedure is detailed in Equation 1.

**Table 1:** Landsat-8 satellite sensor parameters.

Band settings	Wavelength range (nm)	Spatial resolution (m)
Coastal band	433-453	30
Blue band	450-515	30
Green band	525-600	30
Red band	630-680	30
NIR	845-885	30
SWIR1	1560-1660	30
SWIR2	2100-2300	30
Cirrus band	1360-1390	30

**Table 2:** Sentinel-2A satellite sensor parameters.

Band settings	Centre wavelength (nm)	Spatial resolution
Coastal band	443	60
Blue band	490	10
Green band	560	10
Red band	665	10
RE_1_	705	20
RE_2_	740	20
RE_3_	783	20
NIR_1_	842	10
NIR_2_	865	20
Water vapor band	945	60
Cirrus band	1375	60
SWIR_1_	1610	20
SWIR_2_	2190	20

**Table 3:** GF-6 satellite sensor parameters.

Band settings	Wavelength range (nm)	Spatial resolution (m)
Blue band	450-520	16
Green band	520-590	16
Red band	630-690	16
NIR	770-890	16
RE1	690-730	16
RE2	730-770	16
PE	400-450	16
YE	590-630	16


(1) $$\rho_{band}=\frac{\int_{band_{\lambda \;\min}}^{band_{\lambda \;\max}}s_{band}\left(\lambda \right)\rho \left(\lambda \right)d\lambda }{\int_{band_{\lambda \min}}^{band_{\lambda \max}}s_{band}\left(\lambda \right)d\lambda }$$

where $\rho_{band}$ is the simulated satellite band reflectance, $s_{band}\left(\lambda \right)$ is the spectral response function of Landsat−8, Sentinel−2, and GF−6 (**Figure 2**), ${\text{band}}_{\lambda \;\max}$ and ${\text{band}}_{\lambda \min}$ are the upper and lower limits of the band, and ρ(λ) is the measured canopy hyperspectral data.

#### 2.3.2 Screening sensitive bands for apple tree nitrogen content from different satellite simulation data

Due to its normalized construction approach, the Normalized Difference Vegetation Index (NDVI) is relatively sensitive to changes in soil background and can effectively suppress radiometric distortions caused by various environmental factors, including sensor calibration errors, variations in solar elevation angle, terrain fluctuations, cloud shadows, and atmospheric conditions. As a result, NDVI exhibits high stability and reliability in extracting vegetation phenological information (Rokni and Musa, 2019; Zhao and Qu, 2024).

Compared with other commonly used spectral feature extraction methods, such as raw reflectance, first-order derivatives, or simple ratio indices, the normalized vegetation index demonstrates greater robustness under diverse environmental conditions. Although ratio-based indices and derivative spectra can enhance the sensitivity to certain physiological characteristics of vegetation in specific spectral regions, they are more susceptible to noise and atmospheric disturbances, leading to lower stability. In contrast, NDVI, by emphasizing relative differences between spectral bands, effectively mitigates the influence of these external interferences.

Based on these advantages, this study selected the normalized vegetation index as a more stable and adaptable spectral feature construction method to identify spectral bands sensitive to apple tree nitrogen content under different phenological stages and sensor conditions, as shown in Equation 2. Specifically, the Pearson correlation coefficient was employed to preliminarily identify sensitive spectral bands by evaluating the correlations between the NDVI constructed from simulated data of three sensors during various phenological stages and the measured nitrogen content. Subsequently, Partial Least Squares Regression (PLSR) models were constructed using various combinations of the selected sensitive bands to further explore the impact of different band combinations derived from simulated multi-sensor data on the inversion accuracy of apple tree nitrogen content.


(2) $$r_{j}=\frac{\sum_{i}\left[(x_{ij}-{\bar{x}}_{j})\left(y_{i}-\bar{y}\right)\right]}{\sqrt{\left[\sum_{t}{\left(x_{ij}-{\bar{x}}_{j}\right)}^{2}\right]}\left[\sum_{t}{\left(y_{i}-\bar{y}\right)}^{2}\right]}$$

where $\;x_{ij}$ represents the spectral reflectance of the jth wavelength in the ith sample (*i=1,2,…, m, j=1,2,…, n*), $y_{i}$ represents the nitrogen content of the ith sample, ${\bar{x}}_{j}$ is the sample average of $x_{j}$ , ${\bar{y}}_{j}$ is the sample average of $y_{j}$ .

#### 2.3.3 Screening the optimal model for apple tree nitrogen content estimation

A total of 100 samples were collected, from which 60 samples were randomly selected as the training set, and the remaining 40 samples were used as the validation set. The full-band information from different satellite-simulated data was used as independent variables, and the nitrogen content of apple trees was taken as the dependent variable. Two nonlinear machine learning algorithms—Support Vector Machine (SVM) and Backpropagation Neural Network (BPNN) —were employed to construct inversion models of apple tree nitrogen content across different phenological stages. The coefficient of determination (R²) and root mean square error (RMSE) were used to evaluate the accuracy of the models, with the aim of identifying the optimal inversion model and determining the most suitable satellite sensor for estimating apple tree nitrogen content. The calculation formulas for R² and RMSE are provided in Equations 3 and 4, respectively.


(3) $$R^{2}=\frac{\sum_{i=1}^{n}{\left({\hat{y}}_{i}-\bar{y}\right)}^{2}}{\sum_{i=1}^{n}{\left(y_{i}-\bar{y}\right)}^{2}}$$

(4) $$RMSE=\sqrt{\frac{1}{n}{\sum_{i=1}^{n}(y_{i}-{\hat{y}}_{i})}^{2}}$$

where $y_{i}$ is the measured value of canopy nitrogen content, ${\hat{y}}_{i}$ is the predicted value of nitrogen content, $\bar{y}$ is the average of the measured values, and n is the number of samples.

## 3 Results and analysis

### 3.1 Analysis of apple tree canopy nitrogen content in different phenological periods

**Figure 3:**
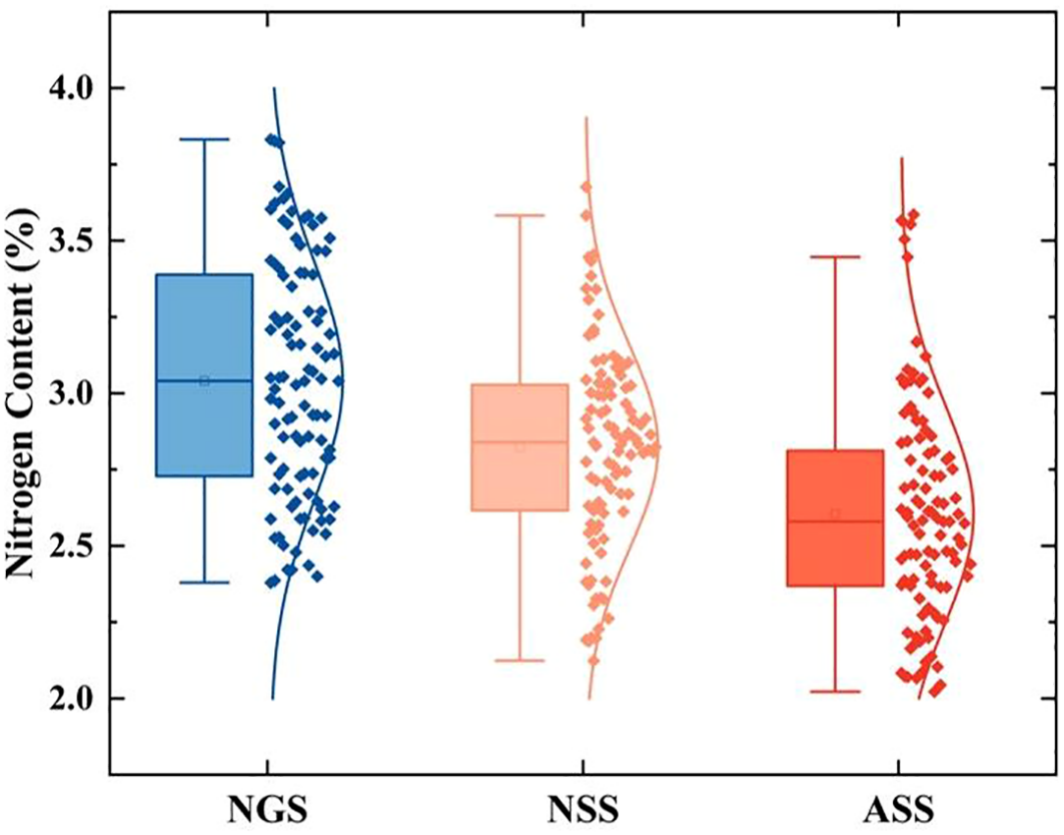
Statistical values of nitrogen content in the canopy of apple trees at different phenological periods.

**Figure 3** shows the changes in apple tree canopy nitrogen content during different phenological periods. From the new shoot growing period to the autumn shoot stop-growing period, the average nitrogen content values were 3.04, 2.82, and 2.60 mg·g^-1^, respectively. The maximum value was 3.83 mg·g^-1^, occurring in the new shoot growing period, and the minimum value was 2.02 mg·g^-1^, occurring in the autumn shoot stop-growing period. This indicates a gradually decreasing trend in apple tree canopy nitrogen content.

### 3.2 Analysis of apple tree canopy hyperspectral and satellite simulation data in different phenological periods

**Figure 4:**
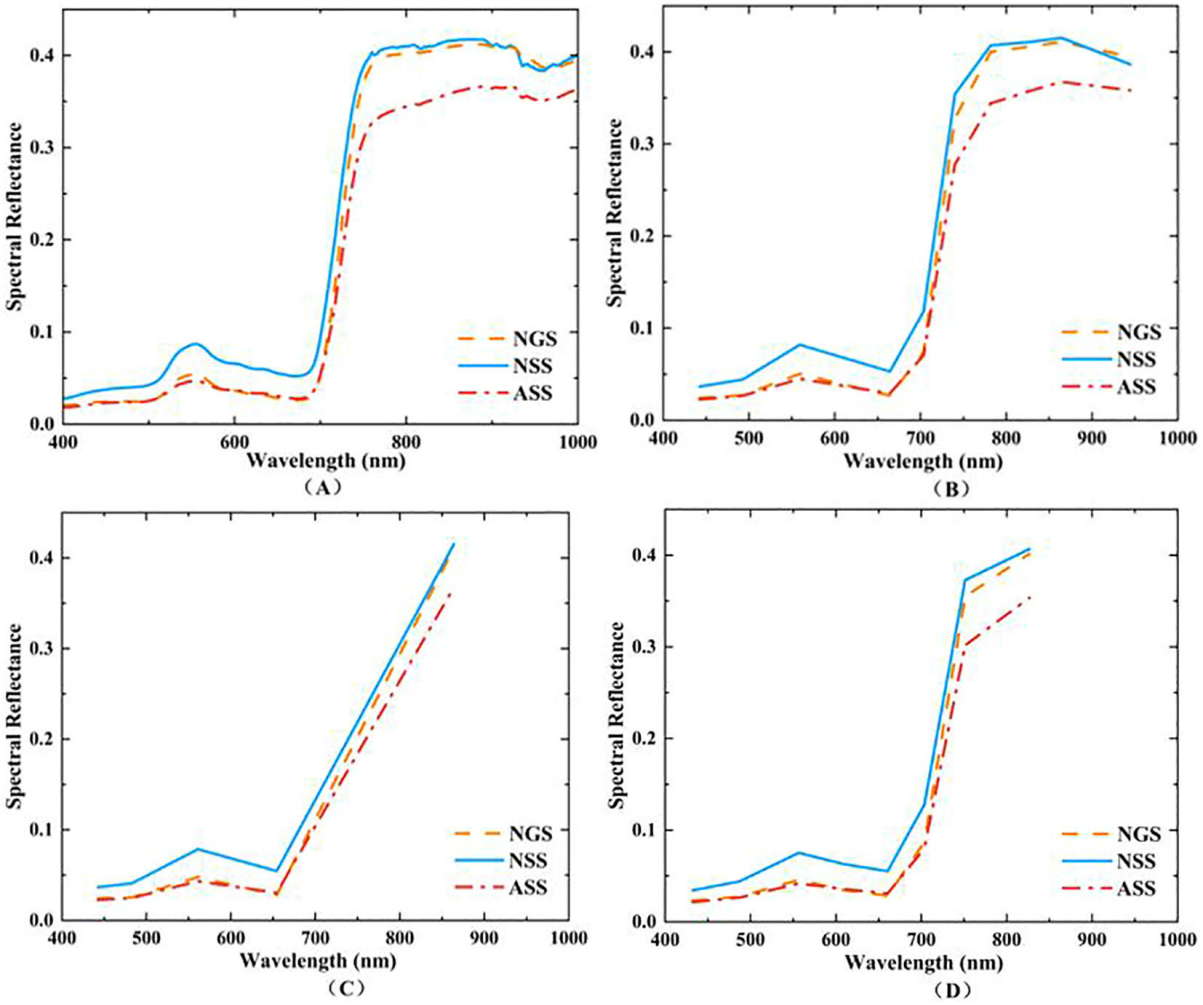
Spectral characteristic curves of apple tree canopy hyperspectral and simulated Landsat-8, Sentinel-2, and GF-6 satellite data in different phenological periods. **(A)** shows the hyperspectral data of the apple tree canopy; **(B)** shows the simulated Landsat-8 data of the apple tree canopy; **(C)** shows the simulated Sentinel-2 data of the apple tree canopy; **(D)** shows the simulated GF-6 data of the apple tree canopy.

The apple tree canopy hyperspectral curves for the three phenological periods are shown in **Figure 4A**. From **Figure 4A**, it can be seen that the canopy spectral curves for the three phenological periods have basically consistent trends and characteristics. Comparing the canopy spectral reflectance values of apple trees in different phenological periods, the canopy spectral reflectance was highest in the new shoot growing period, slightly lower in the spring shoot stop-growing period, and lowest in the autumn shoot stop-growing period. This indicates that the phenological period is an important factor to consider when estimating apple tree canopy nitrogen content.

Based on the spectral response functions of Landsat-8, Sentinel-2, and GF-6, the near-ground hyperspectral data was resampled to obtain simulation data for the three satellite sensors, as shown in **Figures 4B-D**. All three satellite sensors include blue, green, red, and near-infrared bands. Among them, Sentinel-2 is equipped with three red edge bands, while GF-6 includes two red edge bands and one yellow edge band. Among these three sensors, Sentinel-2 has ten bands, GF-6 has eight bands, and Landsat-8 has five bands.

### 3.3 Correlation analysis between different satellite simulation data and apple tree nitrogen content

**Figure 5:**
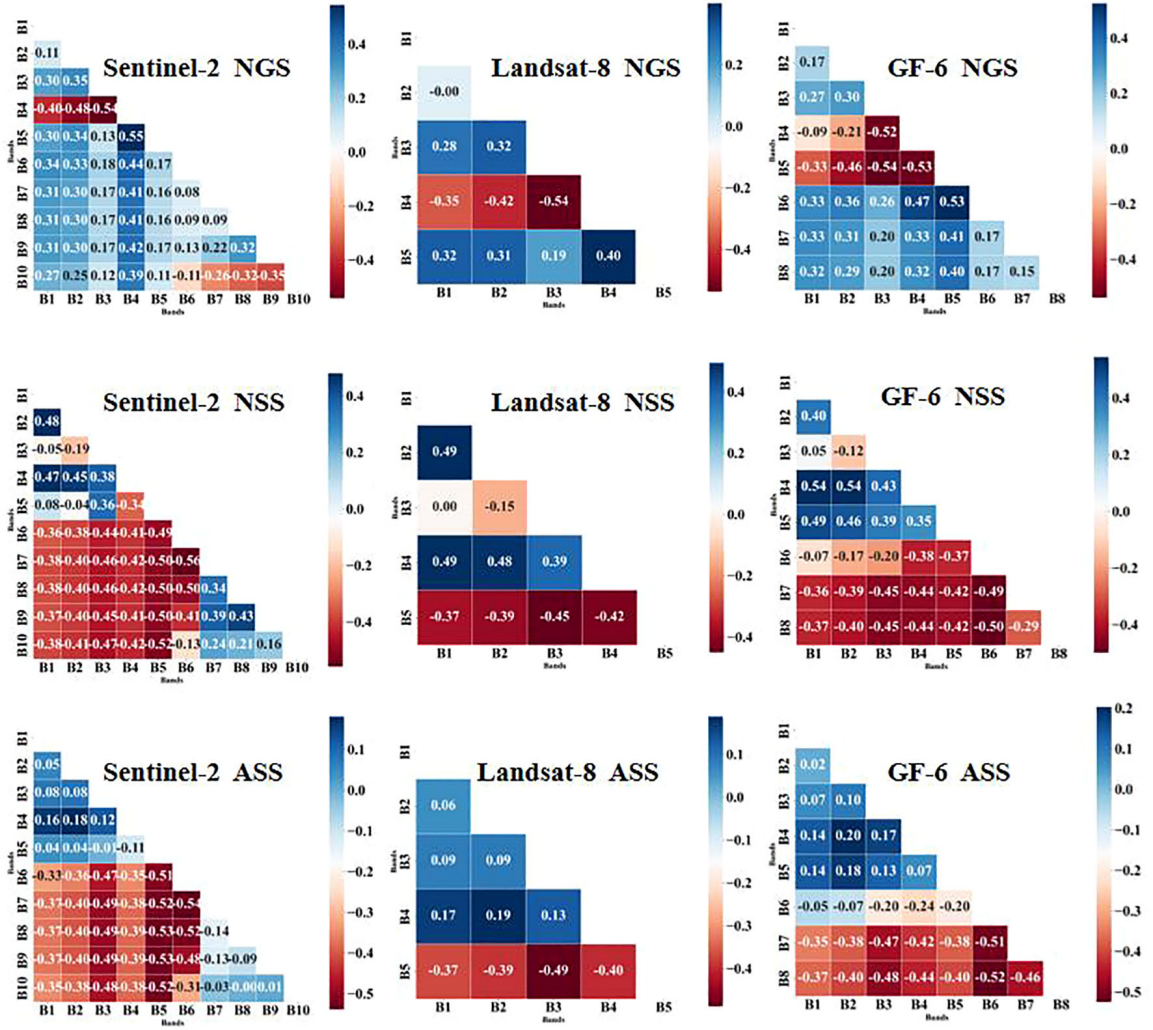
Correlation analysis between different band combinations and nitrogen.

The correlation analysis between normalized vegetation indices and nitrogen content in apple tree canopies (**Figure 5**) revealed that Sentinel-2 imagery consistently demonstrated superior potential for nitrogen monitoring across all three key phenological stages: the new-shoot-growing stage (NGS), the new-shoot-stop-growing stage (NSS), and the autumn shoot-growing stage (ASS). In particular, during the NSS stage, Sentinel-2 achieved a correlation coefficient as high as 0.56, significantly outperforming both Landsat-8 and GF-6. Further investigation indicated that the vegetation indices most strongly correlated with nitrogen content were all derived from Sentinel-2 data. Specifically, the optimal index during the NGS stage involved the combination of the RedEdge1 and NIR bands, while RedEdge2 and RedEdge3 combinations were most effective during the NSS and ASS stages. These findings highlight the pivotal role of red-edge bands in the remote sensing–based assessment of nitrogen status in apple orchards.

From the perspective of spectral response mechanisms, chlorophyll and nitrogen content are the primary factors influencing the absorption and reflectance properties of plant leaves across the electromagnetic spectrum, particularly in the red, red-edge, and near-infrared (NIR) regions. During the NGS stage, the optimal spectral band combinations spanned the red, green, red-edge, and yellow-edge regions—wavelengths known to be highly sensitive to variations in chlorophyll concentration and leaf cellular structure. These bands effectively capture photosynthetic activity and nutrient dynamics during peak vegetative growth. In the NSS stage, the optimal combination expanded to include the blue and NIR bands, likely reflecting structural changes such as increasing leaf thickness and internal anatomical alterations. The blue band is particularly responsive to surface scattering properties, while the NIR band is indicative of the integrity and organization of internal cell structures. By the ASS stage, the most informative bands were primarily located in the red-edge and NIR regions, coinciding with the physiological senescence of the trees, characterized by declining chlorophyll levels and cellular degradation. The strong sensitivity of red-edge and NIR bands to these degenerative processes underpins their effectiveness in monitoring canopy nitrogen status during the later stages of the growth cycle.

### 3.4 Screening sensitive bands for apple tree nitrogen content estimation

The preliminarily screened nitrogen content sensitive bands were combined to construct partial least squares regression models to explore the influence of different sensitive bands on apple tree nitrogen content estimation. The model accuracy results are shown in **Table 4**. The PLSR model results based on Landsat-8 data show that the near-infrared band played a key role in all three phenological periods. In the new-shoot-growing stage, the model including red, green, blue, and near-infrared bands had an R² of 0.48, which decreased by 20% after removing the near-infrared band; in the new-shoot-stop-growing stage, the model R² decreased from 0.53 to 0.50 (a 3% decrease); in the autumn shoot-growing stage, it decreased from 0.29 to 0.19 (a 10% decrease). Notably, the model accuracy was lowest for combinations in Sentinel-2 and GF-6 data that did not include near-infrared bands (R² of 0.31 and 0.27, respectively), further confirming the importance of the near-infrared band.

**Table 4:** Partial least squares regression model based on different sensitive band combinations.

Simulated Data Source	Phenological Stage	Band combination	*R^2^*
Landsat-8	NGS	Red, Green, Blue, Nir	0.48
		Red, Green, Nir	0.44
		Red, Blue, Nir	0.40
		Green, Blue, Nir	0.32
		Red, Green, Blue	0.28
	NSS	Red, Blue, Coastal, Nir	0.53
		Red, Blue, Coastal	0.50
		Red, Coastal, Nir	0.45
		Red, Coastal	0.29
	ASS	Red, Blue, Green, Nir	0.47
		Red, Green, Nir	0.43
		Red, Blue, Nir	0.29
		Red, Blue	0.19
Sentinel-2A	NGS	Red, Blue, Green, RE_1_	0.55
		Red, Green, RE_1_	0.52
		Red, Blue, RE_1_	0.51
		Red, Blue, RE_1_	0.44
		Red, Blue, Green	0.31
	NSS	RE_1_, RE_2_, RE_3_, Nir_1_, Nir_3_	0.50
		Red, Blue, RE_1_, Nir_1_	0.61
		Red, Blue, RE_1_, RE_2_, RE_3_	0.55
		Red, Blue, Nir_1_, Nir_2_	0.53
		Red, Blue, Green	0.49
	ASS	RE_1_, RE_2_, RE_3_, Nir_1_, Nir_2_	0.45
		Red, Blue, Green, RE_1_, Nir_1_	0.58
		Red, Blue, Green, Nir_1_	0.47
		Red, Blue, Green, RE_1_	0.40
GF-6	NGS	Red, Green, RE_1_, YE	0.54
		Green, RE_1_, YE	0.49
		Red, Green, RE_1_	0.47
		Red, Green, YE	0.46
		Red, Green	0.27
	NSS	PE, Blue, YE, RE_1_, Nir	0.57
		PE, Blue, RE_1_, Nir	0.50
		PE, Blue, YE, Nir	0.49
		PE, YE, RE_1_, Nir	0.49
		PE, Blue, Nir	0.44
	ASS	Green, RE_1_, RE_2_, Nir	0.56
		RE_1_, RE_2_, Nir	0.48
		Green, RE_1_, RE_2_	0.43
		Green, Nir	0.40

The PLSR model for Sentinel-2 data indicated that removing red edge bands would result in a significant decrease in model accuracy: a 24% reduction in R² (from 0.55 to 0.31) in the new-shoot-growing stage, a 12% reduction (from 0.61 to 0.49) in the new-shoot-stop-growing stage, and an 11% reduction (from 0.58 to 0.47) in the autumn shoot-growing stage. GF-6 data showed a similar trend, with R² decreasing by 8%, 8%, and 16% in the new-shoot-growing stage, new-shoot-stop-growing stage, and autumn shoot-growing stage, respectively, after removing red edge bands. The yellow edge band unique to GF-6 data showed unique predictive value. Removing this band caused the model R² to decrease by 7% (from 0.54 to 0.47) in the new shoot growing period and by 7% (from 0.57 to 0.50) in the spring shoot stop-growing period.

In addition, all different satellite remote sensing data indicated that the three visible light bands - blue, green, and red - had important influences on estimating apple tree nitrogen content in different phenological periods. In summary, visible light bands, red edge bands, near-infrared bands, and yellow edge bands are important for estimating apple tree nitrogen content.

### 3.5 Screening the optimal inversion model for apple tree nitrogen content estimation

Using all bands of the three satellite simulation data, SVM and BPNN were used to construct apple tree nitrogen content estimation models to screen the optimal satellite for apple tree nitrogen content estimation. The accuracy evaluation results are shown in **Table 5**. A comparison of inversion models constructed using data from different satellite sensors showed that the accuracy of the BPNN model was consistently lower than that of the SVM model across all phenological stages.

**Table 5:** Accuracy evaluation results of SVM and BPNN models constructed using all bands of different simulation data.

Sensor Type	Model Type	Sample Set	Evaluation Metrics	NGS	NSS	ASS
Landsat-8	SVM	Training Set	R^2^	0.64	0.66	0.62
			RMSE	0.21	0.18	0.23
		Validation Set	R^2^	0.51	0.53	0.50
			RMSE	0.34	0.27	0.24
	BPNN	Training Set	R^2^	0.57	0.65	0.60
			RMSE	0.22	0.18	0.23
		Validation Set	R^2^	0.48	0.50	0.59
			RMSE	0.35	0.28	0.24
Sentinel-2	SVM	Training Set	R^2^	0.81	0.83	0.79
			RMSE	0.15	0.12	0.17
		Validation Set	R^2^	0.61	0.63	0.60
			RMSE	0.26	0.23	0.21
	BPNN	Training Set	R^2^	0.77	0.80	0.77
			RMSE	0.17	0.13	0.18
		Validation Set	R^2^	0.59	0.60	0.56
			RMSE	0.28	0.24	0.22
GF-6	SVM	Training Set	R^2^	0.80	0.82	0.78
			RMSE	0.16	0.13	0.18
		Validation Set	R^2^	0.60	0.62	0.61
			RMSE	0.30	0.23	0.21
	BPNN	Training Set	R^2^	0.78	0.78	0.74
			RMSE	0.16	0.14	0.20
		Validation Set	R^2^	0.58	0.64	0.56
			RMSE	0.30	0.22	0.22

**Figure 6:**
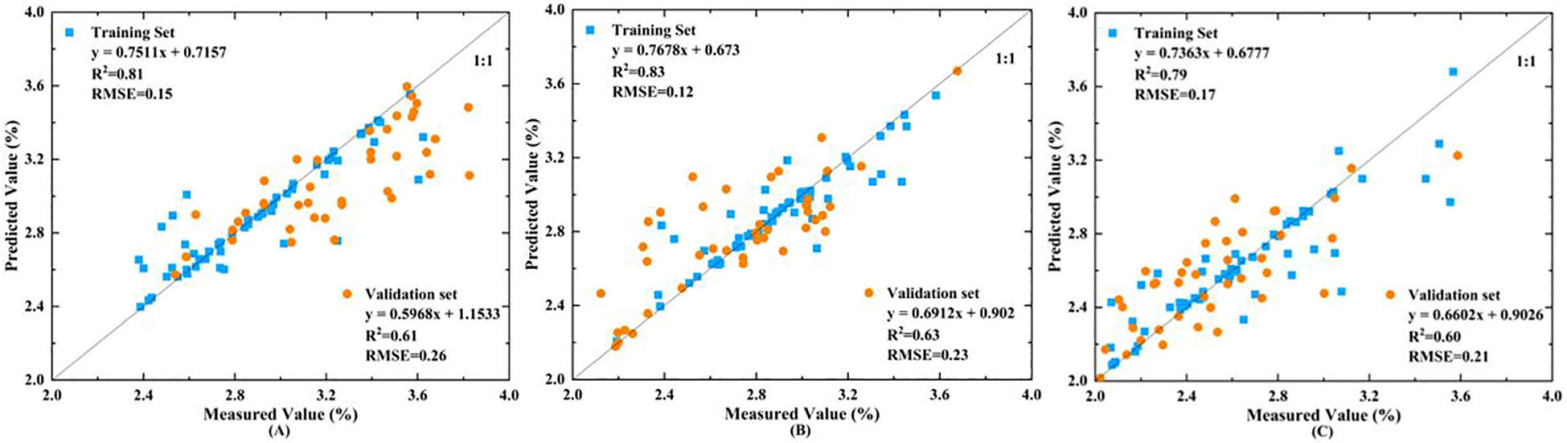
SVM inversion scatter plots for nitrogen content using Sentinel-2 data at different phenological stages. **(A)** NGS, **(B)** NSS, **(C)** ASS.

Comparing the modeling accuracy of the three simulation data, it was found that the support vector machine model constructed based on Sentinel-2 simulation data had an average R² value of 0.81 and an average RMSE value of 0.15 for the training set, and an average R² value of 0.61 and an average RMSE value of 0.23 for the validation set. In contrast, the support vector machine model established based on Landsat-8 bands had an average R² value 0.17 lower than Sentinel-2 and an RMSE 0.06 higher for the training set, and an R² 0.1 lower and an RMSE 0.05 higher for the validation set. Comparing the model accuracy constructed based on Sentinel-2 and GF-6 simulation data, the results showed that the inversion accuracy of Sentinel-2 bands was slightly higher than that of GF-6. In summary, Sentinel-2 satellite simulation data performed best in the application of apple tree nitrogen content estimation. The scatter plots of the optimal inversion models for different phenological periods of apple trees are shown in **Figure 6**.

## 4 Discussion

### 4.1 Impact of phenological period on apple tree nitrogen content estimation

Research has found that from the new shoot growing period to the autumn shoot stop-growing period, apple tree canopy nitrogen content continuously decreases. Apple trees have the highest nitrogen content during the new organ construction period, and as the organs grow, the nitrogen content in the tree body gradually decreases. The main reason is that as the seasons change, nitrogen in apple trees continuously transfers to other new organs. During the new shoot growing period, nitrogen absorbed by the apple tree root system continuously transfers to new organs to meet the nitrogen needs for constructing new shoots and leaves; while in the spring shoot stop-growing period, leaf nitrogen reaches a relatively stable state; after entering the autumn shoot stop-growing period, nitrogen absorbed by the apple tree root is prioritized for root growth, relatively weakening the transport to the upper part of the apple tree (Zhao et al., 2024). Therefore, as shown in **Figure 3**, apple tree canopy nitrogen content shows a decreasing trend from the new shoot growing period to the autumn shoot stop-growing period.

### 4.2 Analysis of sensitive bands for apple tree nitrogen content

There are differences in sensitive bands for estimating apple tree nitrogen content in different phenological periods. However, through correlation analysis of different band combinations with nitrogen content, it was found that the optimal bands within the same phenological period showed certain similarities. Comprehensive analysis of the three satellite sensors’ results showed that during the new shoot growing period, red, green, red edge, and yellow edge bands showed higher sensitivity to nitrogen content; during the spring shoot stop-growing period, the optimal bands were blue, red edge, near-infrared, and yellow edge bands; while during the autumn shoot stop-growing period, red edge and near-infrared bands became the key feature bands (Li et al., 2014; Sun et al., 2021; Zhang et al., 2023; Hou et al., 2024).

Although the optimal band combinations differed across phenological periods, in all three periods, the band combinations with the highest correlation to nitrogen content all came from Sentinel-2, specifically NDVI(Rededge_1_/Red), NDVI(Rededge_2_/Rededge_3_), and NDVI(Rededge_2_/Rededge_3_). This result further confirms the importance of red edge bands in nitrogen estimation. Furthermore, as shown in **Table 1**, the model accuracy established with optimal combinations after removing red edge bands significantly decreased, again emphasizing the key role of red edge bands in nitrogen estimation (Peng et al., 2021). The partial least squares models constructed with different band combinations also verified the important role of near-infrared bands in nitrogen estimation (Jang et al., 2024). Additionally, the study found that yellow edge bands also have important value for nitrogen estimation, indicating that compared to previous high-resolution satellite series, the newly added red edge and yellow edge bands in the GF-6 satellite have significant advantages in vegetation nitrogen monitoring. These findings provide important theoretical basis and technical support for optimizing apple tree nitrogen monitoring.

### 4.3 The optimal satellite for estimating nitrogen content in apple trees and analysis of their interactions

To comprehensively evaluate the capability of different sensors in estimating nitrogen content, we constructed inversion models using support vector machines (SVM) and backpropagation neural networks (BPNN) based on all spectral bands of the three sensors. The results indicate that Sentinel-2 consistently exhibits the highest correlation and modeling accuracy across all phenological stages, followed by GF-6, with Landsat-8 performing the worst. Sentinel-2’s superior performance is attributed to its narrower red-edge bands (15 nm bandwidth), which enable finer detection of subtle changes in the nitrogen content of the apple canopy (Prey and Schmidhalter, 2019). It is noteworthy that there are significant interactions among phenological stages, spectral band characteristics, and sensor configurations. The distribution and variation of canopy nitrogen during different phenological periods affect the sensitivity to specific bands, which vary among sensors. For example, although GF-6 possesses red-edge and yellow-edge bands, its red-edge bandwidth is wider than that of Sentinel-2, resulting in less precise responses to nitrogen variations. This matching relationship between band configuration and physiological changes is a critical factor influencing the accuracy of nitrogen estimation. Therefore, integrating phenological dynamics, spectral band sensitivity, and sensor characteristics contributes to a more comprehensive understanding of the spatiotemporal dynamics of nitrogen in fruit trees and provides a theoretical basis for optimizing remote sensing monitoring strategies.

## 5 Conclusion

Using a method combining near-ground measured canopy hyperspectral data with multi-source satellite simulation, this study evaluated the potential of different open-source satellite remote sensing data in estimating apple tree nitrogen content. Results showed that visible light, red edge, near-infrared, and yellow edge bands are important for apple tree nitrogen estimation. Compared to Landsat-8 and GF-6, the apple tree nitrogen content estimation model constructed using Sentinel-2 satellite remote sensing data was the optimal inversion model. This study provides scientific basis and technical reference for the application of different satellite sensors in apple tree nitrogen monitoring, which is significant for optimizing apple tree nutrient monitoring management. Future research can expand on this study by integrating multi-temporal and multi-source remote sensing data to improve the temporal resolution and robustness of nitrogen content estimation based on Sentinel-2 imagery. Furthermore, the adoption of more advanced machine learning algorithms holds promise for enhancing model performance, thereby advancing fruit tree nutrient monitoring toward greater accuracy and broader applicability.

## Data Availability

The raw data supporting the conclusions of this article will be made available by the authors, without undue reservation.
